# Steering nitrogen emission from poultry production through precision nutrition

**DOI:** 10.1016/j.psj.2026.106864

**Published:** 2026-03-26

**Authors:** Jae Cheol Kim, Behnam Saremi, Wölfgang Siegert, Rommel Sulabo, Sanami Tatekura

**Affiliations:** a CJ Bio, CJ Cheiljedang Corp, Seoul, South Korea; b CJ Bio Europe GmbH, Frankfurt am Main, Germany; c Department of Animal Sciences, University of Göttingen, Göttingen, Germany; d Institute of Animal Science, University of the Philippines, Los Baños, Laguna, Philippines; e AB Vista Asia, Singapore

**Keywords:** Broilers, Crude protein, Precision nitrogen nutrition

## Abstract

Efficient poultry production depends on the optimal supplementation of energy and standardized ileal digestible (**SID**) amino acids to realize the genetic potential for whole-body protein deposition. While the requirements for energy and SID amino acids—such as the SID lysine-to-energy ratio—are well-documented, industry adoption of these findings remains slow. Consequently, many commercial formulations still rely on crude protein (**CP**) as a primary constraint. This reliance on high levels of protein meal inclusion leads to increased indigestible protein and excess absorbed amino acids, which in turn trigger various metabolic, health, welfare, and environmental challenges. This symposium paper explores how the poultry industry can effectively manage nitrogen emissions while maintaining optimal growth performance by leveraging the latest advancements in precision nitrogen (**N**) nutrition.

## Highlights

• Reducing protein with optimal amino acid supplementation does not compromise broiler growth.
• Reducing crude protein effectively mitigates the environmental footprint.
• Lower protein levels improve gut health and animal welfare.
• The successful implementation of these strategies relies on advanced analytical tools.

## Background

Since the 1960s, leaders in academia and industry have progressively refined protein nutrition strategies, moving from crude protein (**CP**) and total amino acids to digestible and, eventually, standardized ileal digestible (**SID**) amino acids. This evolution has paved the way for precision nitrogen (**N**) nutrition. Despite these advancements, a significant number of poultry feed producers in the region still rely on CP and total amino acid formulations.

Ongoing research continues to deepen our understanding of N-precision nutrition (Siegert et al., 2025). This approach focuses on meeting the genetic potential for protein deposition by precisely supplementing digestible amino acids, rather than relying on excessive CP or large inclusions of protein meals. Current practices are estimated to result in over 40-50% of dietary N being excreted as uric acid and undigested forms. This contributes to carbon emissions, soil acidification, and water eutrophication (Cappelaere et al., 2021; Strifler et al., 2023).

High protein levels in broiler diets have two negative effects. First, excess undigested protein acts as a fermentable N source for gut pathogens, leading to harmful microbial profiles and possibly harming intestinal health. Second, while digested and absorbed, surplus amino acids—mainly non-essential ones—must be metabolized and excreted as uric acid. This process increases the energy needed for maintenance, which is an important economic factor in broiler production. Additionally, forming uric acid uses two water molecules, raising water intake and litter moisture content, both of which are critical for poultry health and welfare.

This paper summarizes a symposium held at the 2025 Pacific Rim Poultry Science Association meeting in Macau, China. The authors offer a comprehensive overview of perspectives on precision N nutrition. This overview results from an in-depth examination of how undigested protein and excess amino acids—produced by high-protein diets—affect growth performance, intestinal health, metabolic efficiency, animal welfare, gut health, and environmental impact. This review seeks to bridge the gap between academic advancements in precision nitrogen nutrition and current industry practices by detailing the economic, animal health, and ecological benefits of formulating diets based on digestible amino acids rather than crude protein. The primary objective of this paper is to provide a comprehensive overview of precision N nutrition in poultry, evaluating how transitioning from crude protein to SID amino acid formulations impacts growth performance, bird welfare, and environmental sustainability.

## Impact of nitrogen emission from poultry production on global warming and sustainability

### Behnam Saremi

The Earth is under increasing pressure due to the overutilization of natural resources and the continuous rise in atmospheric carbon dioxide concentrations (NOAA Global Monitoring Laboratory, 2025; Fig.. 1). The livestock industry is essential to produce high-quality and affordable protein, yet it contributes to climate change through both direct and indirect greenhouse gas emissions (Zhang et al., 2023). Feed raw materials are a major driver of environmental impacts in livestock systems. In broiler production, feed accounts for approximately 92%, 76%, and 95% of the global warming potential (**GWP**), acidification, and eutrophication, respectively (Cesari et al., 2017). Excess dietary protein, imbalanced amino acid profiles in feed formulations, or the use of protein sources with low digestibility result in inefficient N utilization (Njeri et al., 2026). Poultry primarily excrete N in the form of uric acid (Creek and Vasaitis, 1961), which can subsequently be transformed into ammonia (**NH₃**), nitrous oxide (**N₂O**), and nitrate (**NO₃⁻**), thereby contributing to environmental degradation. Inadequate manure handling and land application further increase the risk of nitrate leaching and gaseous N emissions. Notably, a 1%-point reduction in dietary CP can lead to an approximately 10% reduction in N excretion (Cappelaere et al., 2021). Therefore, nutritional interventions represent an effective strategy to mitigate N-related environmental impacts. Approaches such as amino acid balancing, low CP diets, and the use of locally sourced feed ingredients or raw materials from land use change-free areas can significantly reduce eutrophication and acidification, two key impact categories in life cycle assessment (**LCA**), alongside GWP. Two trials were conducted to evaluate the impact of nutrition on broiler performance and environmental outcomes.

**Fig. 1:**
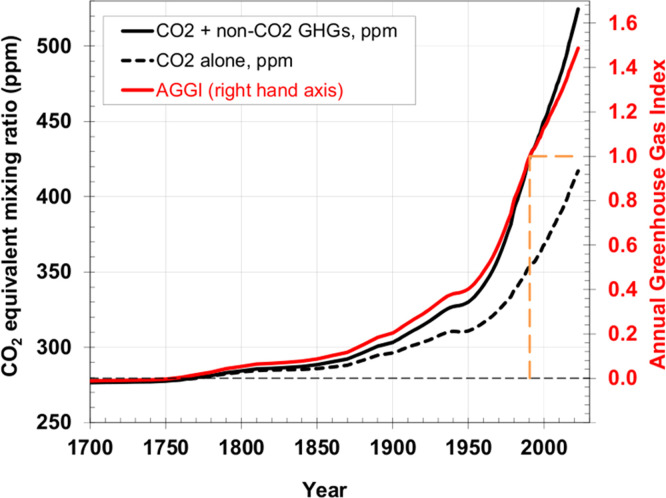
Atmospheric histories since 1700 for CO_2_ abundance (black line), CO_2_-equivalent abundance based on ongoing measurements of all greenhouse gases reported here (black dashed line), and the AGGI (red line, right-hand scale). The measurements of CO_2_ between the 1950s and 1978 are from C.D. Keeling [Keeling, 1958]. Prior to 1978, atmospheric abundances represent values derived from air trapped in ice and snow above glaciers (Machida et al., 1995; Battle et al., 1996; Butler et al., 1999). Equivalent CO_2_ atmospheric amounts (in ppm) are approximated by the relationship between CO_2_ global mole fraction and radiative forcing from all long-lived greenhouse gases (Ramaswamy et al., 2001). The dashed orange lines highlight the AGGI reference year (1990) at which the index is assigned a value of 1.0 (adapted from NOAA Global Monitoring Laboratory, 2025).

### Impact of reducing dietary CP and soybean meal on GWP

Two trials were conducted. Trial 1 was a three-phase broiler study (0–12, 13–21, and 22–35 days) with male Ross 308 birds (*n* = 1350; 225 birds per treatment and 15 birds per pen), allocated to 15 pen replicates per treatment. Dietary CP was reduced in different phases: only in the finisher phase (19% to 17%, T2), in both grower (20% to 19%) and finisher phases (T3), or across all three phases, including starter (21% to 20%, T4). Two additional treatments evaluated soybean meal (**SBM**) inclusion: maximum 15% SBM (T5) or 0% SBM (T6) compared to a positive control (PC; T1), based on which birds were fed according to Aviagen (2019). Body weight (**BW**), daily weight gain (**DWG**), daily feed intake (**DFI**), and feed conversion ratio (**FCR**) were recorded for each phase. On day 36, four birds per pen were used for carcass evaluation. Foot pad lesions (**FPS**) were scored on days 22 and 35. Data were analyzed using a linear regression model, and post-hoc pairwise comparisons were performed without adjustment (*P* < 0.05). Global warming potential was calculated per treatment and growth phase using Opteinics™ (Matton et al., 2025).

Trial 2 evaluated the effects of nutrient recommendations (CVB, 2018) versus breeder guidelines (Aviagen, 2019) corrected for arginine (**Arg**) (115% SID Arg:Lys) and histidine (**His**) (40% SID His:Lys) per CJ Europe GmbH recommendations (CJE, Frankfurt am Main, Germany), combined with low versus normal CP levels in a 2 × 2 factorial design (Table 1, Table 2). A total of 25,920 Ross 308 chickens were allocated to four treatments, with eight replicates of 810 birds each. Birds had ad libitum access to corn–wheat–SBM based diets during four phases: 0–10, 10–22, 22–31, and 31–38 days. BW at arrival was determined by weighing a random sample of 100 birds. During the trial, BW was automatically measured at pen level by weighing platforms. Water intake (**WI**), and feed intake (**FI**) were recorded. Water-to-feed ratio (**WF**) and FCR were calculated. Life cycle assessment (climate change impact) was performed using Opteinics™ by Schothorst Feed Research (SFR, Lelystad, NL). Data were analyzed in Genstat (2023, 23rd Ed.) using two-sided tests, with Tukey post hoc comparisons (*P* < 0.05; van Eerden et al., 2025).

**Table 1:** Feed formulation of different treatments at different growth phases.

	Starter				Grower				Finisher			
	CJ		CVB		CJ		CVB		CJ		CVB	
	Normal CP	Low CP	Normal CP	Low CP	Normal CP	Low CP	Normal CP	Low CP	Normal CP	Low CP	Normal CP	Low CP
Ingredients (%)												
Corn	46.3	54.3	47.7	55.7	44.4	52.6	47.00	54.5	34.7	40.6	38.9	44.6
Soybean meal (>48% CP)	32.5	24.8	33.5	26.7	28.3	20.5	29.3	22.3	23.3	17.5	23.9	18.4
Wheat	7.00	7.00	7.61	7.00	13.00	13.00	13.00	13.00	27.00	27.00	27.00	27.00
Wheat middlings	5.00	5.00	5.00	5.00	5.00	5.00	5.00	5.00	5.00	5.00	5.00	5.00
Soybean oil	3.75	2.23	1.45	0.090	4.59	3.01	1.81	0.450	5.77	4.64	1.60	0.560
Monocalcium phosphate	1.74	1.76	1.72	1.74	1.39	1.41	1.37	1.39	1.11	1.13	1.09	1.11
Limestone (38% Ca)	1.33	1.38	1.32	1.37	0.990	1.04	0.990	1.03	0.880	0.92	0.880	0.920
Salt	0.200	0.040	0.230	0.160	0.200	0.040	0.230	0.120	0.160	0.040	0.200	0.060
Sodium bicarbonate	0.310	0.650	0.270	0.370	0.320	0.580	0.280	0.440	0.380	0.660	0.320	0.560
Choline-Chloride 75%	0.090	0.110	0.080	0.100	0.080	0.110	0.070	0.100	0.070	0.090	0.070	0.080
L-Lysine HCl	0.310	0.550	0.210	0.420	0.310	0.550	0.210	0.420	0.330	0.500	0.260	0.430
DL-Methionine (99%)	0.380	0.440	0.300	0.350	0.340	0.410	0.250	0.310	0.320	0.370	0.220	0.270
L-Threonine (98%)	0.210	0.310	0.080	0.170	0.180	0.290	0.070	0.160	0.160	0.240	0.080	0.160
L-Arginine (99%)	0.140	0.370	-	0.150	0.140	0.370	-	0.160	0.160	0.330	0.010	0.170
L-Valine (96.5%)	0.140	0.270	0.030	0.150	0.120	0.260	0.020	0.140	0.110	0.210	0.030	0.120
L-Isoleucine (90%)	0.080	0.220	-	0.020	0.080	0.220	-	0.030	0.090	0.200	-	0.040
L-Tryptophan (98%)	-	0.030	-	-	-	0.040	-	-	0.010	0.040	-	-
L-Histidine H2O HCl (98%)	-	0.050	-	-	-	0.050	-	-	-	0.040	-	-

CJ: according to Aviagen recommendations (2022) for Ross 308 corrected for standardized ileal digestible (SID) arginine to lysine at 115% and SID histidine to lysine at 40%.

CVB: Dutch recommendations (2018).

Normal crude protein (CP): 217, 201, and 187 g/kg in starter, grower and finisher feed, respectively.

Low CP: 197, 182, and 172 g/kg in starter, grower and finisher feed, respectively.

**Table 2:** Diet composition in starter, grower and finisher feeds.

		Starter				Grower				Finisher			
		CJ		CVB		CJ		CVB		CJ		CVB	
		Normal CP	Low CP	Normal CP	Low CP	Normal CP	Low CP	Normal CP	Low CP	Normal CP	Low CP	Normal CP	Low CP
CP	g/kg	217	197	217	197	202	182	202	181	187	172	187	171
Crude fat	g/kg	63.3	49.7	41.2	29.3	71.7	57.6	45.1	33.1	82.4	72.3	42.6	33.3
Crude fiber	g/kg	25.6	24.5	26.3	25.4	25.2	24.1	26.0	25.0	24.9	24.1	25.8	25.0
Calcium	g/kg	9.13	9.13	9.09	9.11	7.14	7.13	7.13	7.12	6.14	6.16	6.12	6.15
Phosphorus	g/kg	7.72	7.48	7.78	7.59	6.79	6.55	6.86	6.66	6.00	5.84	6.09	5.94
Choline	mg/kg	675	825	600	750	600	825	525	750	525	675	525	600
dEB	meq/kg	253	242	262	228	235	214	243	216	218	213	221	213
ME	kcal/kg	2,931	2,919	2,786	2,778	3,029	3,018	2,860	2,851	3,130	3,121	2,885	2,878
SID lysine	g/kg	12.1	12.1	11.6	11.6	11.2	11.2	10.7	10.7	10.2	10.2	9.88	9.92
SID methionine	g/kg	6.56	6.80	5.85	6.05	5.98	6.32	5.18	5.47	5.57	5.80	4.67	4.92
SID cysteine	g/kg	2.58	2.31	2.67	2.43	2.48	2.20	2.56	2.32	2.39	2.18	2.48	2.29
SID methionine plus cysteine	g/kg	9.14	9.11	8.52	8.47	8.46	8.53	7.74	7.78	7.96	7.99	7.15	7.21
SID threonine	g/kg	8.47	8.47	7.40	7.43	7.67	7.76	6.79	6.80	6.87	6.92	6.28	6.37
SID tryptophan	g/kg	2.11	2.03	2.18	1.85	1.96	1.97	2.02	1.68	1.92	1.93	1.87	1.60
SID valine	g/kg	9.76	9.76	8.96	9.02	8.95	9.03	8.24	8.28	8.16	8.18	7.63	7.62
SID isoleucine	g/kg	8.43	8.42	7.95	7.01	7.81	7.78	7.32	6.46	7.21	7.24	6.60	6.07
SID leucine		15.1	13.3	15.6				14.5				13.9	11.8
SID arginine	g/kg	13.9	14.0	12.9	12.4	12.8	12.8	11.8	11.4	11.7	11.7	10.6	10.6
SID histidine	g/kg	4.95	4.61	5.10	4.49	4.58	4.23	4.73	4.11	4.14	3.90	4.29	3.79
SID tyrosine	g/kg	6.29	5.31	6.48	5.63	5.76	4.79	5.96	5.09	5.13	4.40	5.31	4.63
SID phenylalanine	g/kg	9.03	7.66	9.32	8.11	8.36	6.97	8.64	7.41	7.60	6.57	7.86	6.90
SID glycine plus serine	g/kg	16.2	13.7	16.7	14.5	15.0	12.5	15.5	13.3	13.7	11.8	14.1	12.4

CJ: according to Aviagen recommendations (2022) for Ross 308 corrected for SID arginine to lysine at 115% and SID histidine to lysine at 40%.

CVB: Dutch recommendations (2018).

Normal crude protein (CP): 217, 201, and 187 g/kg in starter, grower and finisher feed, respectively.

Low CP: 197, 182, and 172 g/kg in starter, grower and finisher feed, respectively.

dEB: dietary electrolyte balance.

ME: Metabolizable energy.

SID: Standardized ileal digestible.

Trial 1 showed that low CP diets had no negative impact on performance parameters, regardless of the duration of low CP feeding. Recent research has demonstrated that reducing dietary CP without affecting performance is possible (Woyengo et al., 2023), although the study period (life phase) may vary and could potentially influence the outcomes. The low SBM group exhibited higher BW and DWG at day 22 compared with the Control group, while both the zero and low SBM groups remained superior to the Control group at day 35, consistent with observations by Van-Hofstraeten (2023). Birds receiving low CP diets only during the grower and finisher phases also showed improved DWG from day 22 to 35 compared with the Control group. Birds in the zero SBM group had a better FCR than the PC during the starter and grower phases, but not during the finisher phase. Over the entire experiment, both the low and zero SBM groups exhibited superior FCR, carcass weight, and breast meat yield compared with the Control group, confirming previous findings (van Harn et al., 2019). Feeding a low CP diet either during the finisher phase or across all three phases also had a positive effect on FPS at day 35. In the starter phase, CO₂-equivalent emissions were 1726, 1548, 1283, and 748 kg per ton of feed for T1–T3, T4, T5, and T6, respectively. In the grower phase, emissions were 1708, 1541, 1342, and 781 kg CO₂ per ton of feed for T1–T2, T3–T4, T5, and T6, respectively. In the finisher phase, emissions were 1612, 1262, 1335, and 810 kg CO₂ per ton of feed for T1, T2–T4, T5, and T6, respectively. The positive impact of SBM reduction depended on the SBM source. Replacement of imported SBM with land use change by local raw materials resulted in a more pronounced reduction in GWP (Van-Hofstraeten, 2023).

Outcomes of trial 2 demonstrated that the treatments formulated according to a modified breeder recommendation outperformed those formulated according to CVB, as expected (Luo et al., 2025; Liu et al., 2019), likely due to higher feed density in terms of AMEn and SID Lys. Low CP feed resulted in a lower BW and FI, but did not affect FCR, likely because the feed lacked certain essential AAs such as phenylalanine and tyrosine, which are not registered in EU or not commercially available. The success of a low dietary CP approach depends on maintaining stable ratios of amino acids (**AAs**) relative to Lys. Moreover, His requirements are not well defined, and Arg requirements in modern broilers are likely higher than assumed in breeder company recommendations for current broiler types (Zampiga et al., 2019; Corzo et al., 2021; Brugaletta et al., 2023; Verhelle and Saremi, 2025; Westreicher-Kristen et al., 2025). However, low CP feeding led to reduced WI and WF.

The climate impact of broiler nutrition has been reviewed with disappointing results (Alkhtib et al., 2023). Of 6142 articles screened, only 29 met the inclusion criteria, with 15 studies including LCA data and the remaining 14 analyzing NH_3_ emissions in broilers. All studies were descriptive and lacked replication. The climate change impact (kg CO_2_ per ton of carcass) of a high-density feed (i.e., breeder recommendations with normal CP) was highest at the feed level but lowest at farming and harvest levels. Low CP feed reduced the LCA impact at the feed level, had a minor effect at the farming level, but had a higher impact at the harvesting level. To our knowledge, this is the first study to evaluate the effect of dietary interventions on LCA using replicated treatments, allowing proper statistical analysis.

Collectively, the two trials demonstrated that low CP diets can be applied to broiler feeding programs without negatively affecting performance parameters. Low and zero-SBM diets demonstrated superior performance and sustainability compared to SBM-based diets. Additionally, birds fed diets formulated according to breeder recommendations exhibited higher BWG and lower FCR, which were not significantly affected by CP level. However, when combined with normal dietary CP, this strategy resulted in the highest impact on climate change. Low CP feeds can therefore be used as a tool to reduce the environmental footprint of poultry meat production, but they may be associated with reduced bird performance if certain essential and non-essential AAs, which are currently not available in feed-grade form, become limiting in low CP diets.

## Impact of crude protein in poultry diet on performance, metabolic efficiency, gut health and welfare

### Jae Cheol Kim

Precision N nutrition shifts broiler feed formulation from total CP content to the requirement for digestible AAs. This approach, which involves reducing dietary CP while precisely balancing SID AA, is essential for minimizing the negative physiological, health, and welfare consequences associated with excess protein. High CP diets lead to two primary issues: the detrimental effects of indigestible protein (**IDP**, defines as total dietary CP – standardized ileal digestible dietary protein; de Oliveira et al., 2025) in the lower gut and the metabolic burden of absorbed but excess AA. By adopting precision N nutrition, broilers exhibit improved N retention, enhanced metabolic efficiency, superior gut health, and reduced risks of welfare issues stemming from poor litter quality.

### Impact of indigestible protein on intestinal health

A significant portion of CP in feed ingredients is indigestible, with poultry CP digestibility varying widely among sources (Rostagno et al., 2024). For example, while blood plasma contains around 10% IDP, feather meal contains around 30% IDP. This IDP passes into the large intestine, where it negatively impacts gut health. Indigestible protein acts as a substrate for microbial fermentation, leading to the production of noxious metabolites such as NH_3_, amine and hydrogen sulfide (**H_2_S**; Davila et al., 2013). Ammonia toxicity in epithelial tissues is well studied. The non-ionized form of NH_3_ penetrates the mitochondrial membrane in enterocytes, increasing mitochondrial pH. This action dose-dependently inhibits mitochondrial respiration, blocking energy synthesis and causing mucosal cell damage (Tsujii et al., 1992; Andriamihaja et al., 2010). Hydrogen sulfide-driven inflammation is also well documented. Excess H_2_​S generates inflammation through multiple pathways. It damages DNA, inhibits DNA methylation, suppresses mucus synthesis, and inhibits butyrate oxidation, thereby creating an energy deficit in colonocytes (Wang et al., 2011; Derdevic et al., 2021). Consequently, high protein diets predispose poultry to enteric diseases, including Necrotic Enteritis (*Clostridium perfringens*), Coccidiosis (*Eimeria* parasites), and *E. coli* infections. Reducing the dietary CP content is a fundamental strategy to reduce IDP, which is shown to negatively impact FCR in broilers (de Lange et al., 2003). Using Ross 308 broiler, a Canadian study demonstrated that a high IDP diet (6.15%) significantly decreased body weight gain (2015 g vs. 1988 g at day 35, *P* = 0.04) and increased FCR (1.47 vs. 1.49, *P* = 0.0015), compared with a low IDP diet (4.49%; Bryan, 2018).

### Impact of absorbed but excess amino acids on physiology and welfare

When CP is excessive, the surplus AA digested and absorbed from the small intestine must be metabolized, leading to detrimental physiological and welfare outcomes. Excess absorbed AA undergo oxidation, leading to a decrease in N retention efficiency. The extent of this catabolism is reflected in higher plasma uric acid N content in birds consuming high protein diets. In poultry, the synthesis of uric acid from AA catabolism is an energy-intensive process. The energy cost is 850 kJ/mol N (equivalent to 9.71 kJ/g excess protein), meaning that 1 gram of excess protein increases the maintenance energy requirement by 2.32 kcal, indicating that conversion of 1% of excess amino acids (i.e., absorbed above the requirement) to uric acid alone could consume 23 kcal apparent metabolizable energy (**AME**) as part of the maintenance energy requirement (van Milgen, 2021). By reducing CP, the maintenance energy required for deamination decreases significantly. This energy conservation increases the AME value of the feed, which has large implication on economy of the commercial broiler feed industry. Previous research confirms that reduced CP diets often lead to a decrease in the energy required for excess AA deamination, frequently increasing abdominal fat pad weight (Hernandez et al., 2025; personal communication with Dr J.T. Lee). In a recent study with Ross 708 broilers, Hernandez et al. (2025) demonstrated that the in vivo measured AME was increased by 45 kcal per each 1% reduction of CP. This excess energy can be utilized either to drive protein accretion (by elevating lysine content) or to reduce overall diet cost.

Reducing CP level in broiler diets can also improve the welfare and sustainability of poultry production. Excess absorbed amino acids lead to increased water consumption and excretion, as water (**H_2_​O**) is required for the formation of uric acid, which is then excreted with water (van Milgen, 2021). This increased water excretion is the primary cause of the 'wet litter' issue. Lemme and his co-workers demonstrated that a reduction of a 2.5% weighted average CP intake (20.4 vs. 17.9% CP) for 40 days reduced litter dry matter content (39.5% vs. 44.5%) and decreased the proportion of birds with foot pad lesions from 75% to 29% (Lemme et al., 2019). According to a meta-analysis study, a 1% reduction in dietary CP can reduce water consumption by 3% or 5 mL water intake per bird (Alfonso-Avila et al., 2022). The global poultry population is estimated to be 26.56 billion (FAO, 2022), and 1% reduction of CP for all poultry diets can save 46.6 billion liters of water annually, which is a significant reduction in water usage for poultry production. Additionally, high protein diets increase the stress response, whereas reduced protein diets can reduce core body temperature as conversion of excess amino acids to uric acid accelerates internal heat production (van Milgen, 2021), offering a physiological advantage, particularly during heat stress.

Precision N nutrition is essential for sustainable and efficient broiler production. Through the strategic use of crystalline amino acids and precise formulation, low-CP diets can maintain or even enhance growth performance while strictly adhering to essential amino acid requirements (Ospina-Rojas et al., 2014; van Harn et al., 2019; Lambert et al., 2023). A meta-analysis by de Rauglaudre et al. (2023) highlighted significant methodological flaws in many studies investigating broiler performance on low CP diets. A primary error identified was the failure to meet requirements for all essential amino acids (**EAA**) within the experimental formulations. When the meta-analysis included all available studies regardless of formulation accuracy, broiler performance on low CP diets plummeted to just 50% of the high CP control group. Conversely, when the dataset was refined to include only those studies that satisfied all EAA requirements—including glycine equivalency (**Gly_equi_**)—the performance deviation was narrowed to within 10% of the high CP-fed birds.

Furthermore, the success of CP reduction depends on the rigorous control of all essential amino acids and the monitoring of dietary electrolyte balance (**dEB**). Maintaining dEB is critical, as values falling below approximately 200 mEq/kg can impair performance, particularly under heat stress in summer months (Lambert et al., 2023). As highlighted by de Rauglaudre et al. (2023), the term 'low-protein diet' lacks precision; broiler responses vary significantly depending on the degree of CP reduction (Siegert and Rodehutscord, 2019; Ibrahim et al., 2024b; Siegert et al., 2025). Broiler performance can typically be maintained when CP is reduced by up to 2% from commercial standards (e.g., breeder recommendations), provided that all essential amino acids are supplied at required levels. Furthermore, performance remains stable with CP reductions of 3% to 4% if both EAA and Gly_equi_ are supplemented (Siegert and Rodehutscord, 2019; Ibrahim et al., 2024b; Siegert et al., 2025). However, further CP reductions often compromise performance despite EAA and Gly_equi_ fortification, suggesting that other nonessential amino acids may be necessary for optimal protein deposition. To address the ambiguity surrounding the term "low-protein diet," such diets should be classified into three distinct categories: (1) Reduced-protein diets: < 2% CP reduction, (2) Moderately reduced-protein diets: 2–4% CP reduction, and (3) Extremely reduced-protein diets: > 4% CP reduction. Establishing this standardized nomenclature will eliminate the confusion currently caused by vague terminology in the literature (Siegert et al., 2025).

Precision nutrition addresses the two primary nutritional drawbacks of conventional high-CP diets: it improves gut health by minimizing IDP-derived noxious compounds (ammonia and H_2_​S), and it enhances metabolic and welfare outcomes by eliminating the high-energy cost of catabolizing excess absorbed AA and mitigating wet litter issues.

### Limitations of low CP diets

While the transition to low-CP diets offers clear advantages for gut health and environmental sustainability, successful implementation requires overcoming several nutritional and physiological hurdles. A primary risk of reducing intact protein in poultry diets is the reduction of the non-essential amino acid pool, particularly Gly+Ser. As CP levels drop, the endogenous synthesis of Gly may become the rate-limiting step for nitrogen excretion, leading to a buildup of toxic ammonia if not supplemented (Siegert and Rodehutscord, 2019). Formulations must now account for the sum of glycine (**Gly**) and serine (**Ser**) to ensure adequate growth and metabolic stability in moderately and extremely reduced CP diets. Secondly, in corn-based diets, the reduction of soybean meal—compensated by crystalline amino acids—often leads to an imbalance among the three branched-chain AAs (**BCAAs**). Excess Leu acts as a potent signal that activates the branched-chain alpha-keto acid dehydrogenase complex. This enzyme complex co-catabolizes all three BCAAs. High dietary Leu can inadvertently trigger a deficiency in valine (**Val**) and isoleucine (**Ile**), even if those amino acids meet NRC or breeder recommendations on paper (Kriseldi et al., 2022). Maintaining ideal BCAA ratios is therefore critical to prevent depressions in feed intake and muscle deposition. Third, while lower CP reduces the heat increment of feeding—providing a theoretical advantage in hot climates—the loss of protein-bound minerals can be problematic. Reducing CP levels often lowers the dietary cation-anion difference, primarily due to less potassium from soybean meal. If the dEB is not corrected, birds may struggle with respiratory alkalosis during panting (Belhadj-Slimen et al., 2016). Finally, future N precision models must account for the fact that SID values are not static; heat stress can alter gut transit time and transporter expression, requiring AA profiles under heat stress.

## Reducing nitrogen emissions through precision N nutrition: what we know so far

### Wolfgang Siegert

Goals of livestock nutrition include minimizing the negative effects of the sector on the environment and contributing to global food security. One way to achieve these goals is to reduce the dietary CP concentration in animal diets without undesirable effects such as health implications and reduced performance. Following this strategy requires a more precise supply of individual amino acids and other nitrogenous nutrients according to situation-specific requirements of the animals. This can reduce an oversupply of nitrogenous nutrients, thus diminishing dietary CP without limiting what is needed by the animals. A key figure to assess the preciseness of the supply of nitrogenous nutrients is the N utilization efficiency (**NUE**), which summarizes the efficiency of the conversion of protein from the feed to animal protein. The NUE is calculated by mass balance method which is estimated via measurement of N intake and N excretion, or a comparative slaughter method which measures the N intake and N retained in the carcass. The typical NUE ranges between 42 and 28% when a traditional high protein diet is fed, while feeding a precision N diet improves the NUE to 60-68% (Cappelaere et al., 2021). This contribution summarized the current status of knowledge on requirements of nonessential amino acids (mostly referring to our own recent review Siegert et al., 2025) and presented insights into a research project on the utilization of peptide-bound vs. free amino acids.

### Requirement for nonessential amino acids

Current applied research focuses on the relevance of essential amino acids. Practical experiments show that reducing dietary CP by 2 to 3 percent units compared to current standard recommendations is feasible without adverse effects on performance (e.g., Cappelaere et al., 2021). The possibility of reducing dietary CP without undesirable effects on performance is limited to 19–20% of feed for up to 21-day-old broiler chickens, even when the requirements for essential amino acids are met (as summarized by Dean et al., 2006). Meanwhile, the nonessential amino acids Gly and Ser, to current knowledge best summarized as Gly_equi_ are known to be the limiting factor when dietary CP is reduced below 19–20%.

**Fig. 2:**
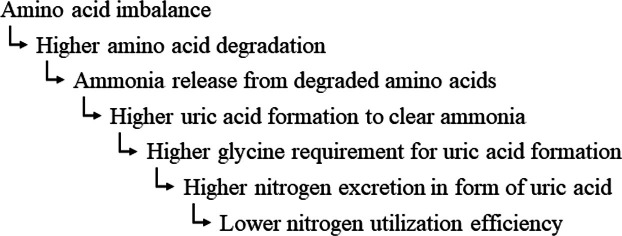
A conceptual schematic of nitrogen utilization efficiency.

The Gly_equi_ requirement is highly variable. Several factors influencing the Gly_equi_ requirement were determined. The fit between supply and requirement for digestible amino acids seems to be the major determinant of the Gly_equi_ requirement. The physiological background is that amino acids that cannot be used for metabolic functions are oxidized and the contained N is excreted via the urine, mostly as uric acid. As uric acid formation dissipates Gly, excess dietary amino acids relative to what can be utilized increases the Gly_equi_ requirement. The Gly_equi_ requirement also increases when one or more amino acids limit protein synthesis because the other amino acids would then be supplied in relative excess and cannot be utilized for protein synthesis (Fig. 2). A model calculation of Siegert and Rodehutscord (2019) suggested that uric acid formation is a major contributor to variable Gly_equi_ requirements. Selle et al. (2021) quantified the proportion of Gly intake for uric acid synthesis in the range of 25–81%. Recalculations of data from our own earlier published studies showed proportions of Gly_equi_ intake relative to uric acid excretion with ranges of 8–30% (Hofmann et al., 2019) and 9–19% (Hofmann et al., 2020a). The higher magnitude of the values of Selle et al. (2021) compared to Hofmann et al. (2019) and Hofmann et al. (2020b) is no contradiction because Selle et al. (2021) referred to Gly while the other studies used Gly_equi_. Regardless of this methodological difference, all 3 studies demonstrate the high amount of dissipated Gly for uric acid synthesis in relation to the dietary Gly and Ser supply. Notably, more Gly_equi_ than the dietary intake was probably available for metabolic purposes than the amount of ingested Gly that was not used for uric acid synthesis in those studies, since both Gly and Ser were probably formed endogenously.

There are other influences on the Gly_equi_ requirement, but those probably have a low impact when compared to uric acid formation. Such influences include dietary threonine and choline because Gly or Ser can be formed in the metabolism of the animals from these compounds (Meléndez-Hevia et al., 2009). The ratio of methionine to cysteine also impacts the Gly_equi_ requirement due to the endogenous conversion of methionine to cysteine, which dissipates Ser. Studies have shown that the impacts of threonine, choline, and the methionine to cysteine ratio on the Gly_equi_ requirement are inconsistent. Some studies found a considerable impact of the mentioned factors on responses to dietary Gly_equi_ (Corzo et al., 2009; Siegert et al., 2015) while others reported minor (Chrystal et al., 2020) or no impact (Hofmann et al., 2020a). One possible contributor to the absence of interaction effects of dietary threonine and Gly_equi_ on performance is a relatively low variation in dietary threonine and Gly_equi_ supply and an overall high Gly_equi_ supply. These characteristics may have made interaction effects undetectable. However, variation of dietary Gly_equi_ and the methionine to cysteine ratio was considerable in the study by Hofmann et al. (2020a), yet resulting in interactions of performance traits of low extent. The NUE was very high at approximately 80% and the variation in NUE was small in the study by Hofmann et al. (2020a). This probably resulted in a low Gly_equi_ requirement of the broiler chickens that was barely influenced by uric acid formation. The impact of interactions between dietary Gly_equi_ and the other mentioned factors most likely represented a mixed consequence of the respective physiological aspect under investigation and an affected NUE, which leads to variable uric acid formation. Most of the mentioned studies did not report NUE. Given the big impact of NUE/uric acid formation on the Gly_equi_ requirement (Siegert and Rodehutscord, 2019; Selle et al., 2021), the small interaction effects in the study of Hofmann et al. (2020a), where NUE was almost unaffected, suggest that other impacts on Gly_equi_ requirements than NUE/uric acid formation have little impact.

Meeting adequate dietary concentrations of Gly_equi_ in addition to essential amino acids allows to reduce dietary CP considerably below the 19–20% in the first 21 days of age, without considering Gly_equi_. Hofmann et al. (2019) determined higher growth of 8–21-day-old broiler chickens than the breeder’s recommendations of the time (Aviagen, 2014), fed with a diet containing 16.3% CP and adequate essential amino acids, as well as Gly_equi_ contents. Further lowering dietary CP to 14.7% by reducing the contents of nonessential amino acids other than Gly_equi_ reduced growth. The conclusion by the authors was that other nonessential amino acids became growth-limiting when CP was reduced from 16.3% to 14.7%. Those findings are supported by Chrystal et al. (2020), who found that the growth of 14–35-day-old broiler chickens was not reduced when dietary CP in Gly-supplemented diets was lowered to 16.5%, which was the lowest CP content in that study. Impacts of such a low dietary CP content without impaired growth on the NUE compared to CP contents currently common in the industry are big. The 16.3% CP diet investigated by Hofmann et al. (2019) resulted in an NUE of 75%, and the NUE determined in newer own experiments found values of above 80% (Hofmann et al., 2020a; 2020b). Such high levels of NUE leave much less N in the excreta that can cause nitrogenous emissions compared to currently common dietary CP levels.

### Utilization of free and peptide-bound amino acids

Absorption processes in the intestine differ between peptide-bound and free amino acids. When peptide-bound amino acids are fed, ingested amino acids are mainly absorbed into the enterocytes after digestion to di- and tripeptides. Such peptides are hydrolyzed and the amino acids are either oxidized in the enterocytes or transferred to the systemic circulation. Dietary free amino acids are directly available for transport to the systemic circulation. Studies on several species have shown that free amino acids are absorbed more rapidly into the systemic circulation than peptide-bound amino acids (e.g., Zamani et al., 2021, Eugenio et al., 2023). With different absorption rates, amino acids are often hypothesized to be available in the systemic circulation in an unbalanced pattern for protein synthesis, even though dietary amino acid supply is formulated as balanced (Namroud et al., 2010). This would result in increased amino acid oxidation and a reduced NUE. However, the different digestibility of free and peptide-bound amino acids was not considered in such studies. A possible relevance of amino acid digestibility was indicated in studies investigating the postprandial appearance of amino acids in the systemic circulation. In such studies, the area under the curve was higher for free compared to peptide-bound amino acids, in addition to the earlier appearance (e.g., Zamani et al., 2021, Eugenio et al., 2023). This may have caused the outcomes of such studies to be not only determined by the time of appearance in the systemic circulation but also by different quantities of amino acids being available for metabolic purposes, including oxidation.

**Fig. 3:**
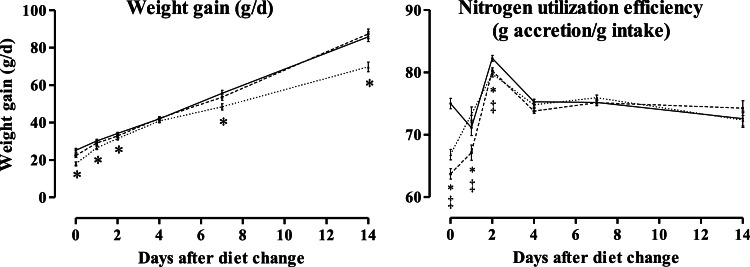
Effects of substitution of the digestible concentration of the 20 proteinogenic amino acids in soy protein isolate with free amino acids, resulting in 19 (solid line), 54 (dashed line), and 87 (dotted line) g free amino acids/kg. Asterisks and crosses indicated that 54 and 87 g free amino acids/kg treatments differed significantly (*P* < 0.05) from 19 g free amino acids/kg for the respective day after diet change. Further information on the experiment is described in the publications (Ibrahim et al., 2024a, b, c).

Consequences of the substitution of peptide-bound from 80 g soy protein isolate/kg of diet (as a feed ingredient containing almost exclusively peptide-bound amino acids) with free amino acids were recently investigated in a project. All 20 proteinogenic amino acids were substituted on a digestible basis based on a forerun digestibility experiment of the investigated diets. Results (Fig. 3) showed that 50% substitution (54 g free amino acids/kg) had no effect, while 100% substitution (87 g free amino acids/kg) reduced weight gain and feed intake (Ibrahim et al., 2024bc). However, NUE was unaffected by amino acid substitution after 4 days of adaptation to the diets (Ibrahim et al., 2024c). This, together with further results (Ibrahim et al., 2024a) of this project, indicated that reduced feed intake seemed to be the primary cause for reduced performance at high levels of dietary free amino acids, but the utilization of the ingested N was unaffected after the adaptation time.

Reducing CP concentrations to ∼16% in diets for broiler chickens up to first 3 weeks of age is possible without compromising growth. This requires well-balanced essential amino acids and consideration of variable Gly_equi_ requirements. Economic feasibility will depend on the development of market prices for protein feeds and the cost impact of environmental legislation. A recent project found no indication of a different utilization of free and peptide-bound amino acids up to an inclusion level much higher than the current and foreseeable standard. Nonetheless, affected growth was found at very high inclusion of dietary free amino acids, with reduced feed intake hypothesized as the main cause.

## The role of reduced crude protein diets in antibiotic growth promoter–free poultry production toward enhanced sustainability

### Rommel C. Sulabo

Global poultry production faces increasing pressure to improve sustainability while maintaining productivity and profitability. Rising feed costs, volatile protein ingredient markets, and increasing societal concerns about environmental pollution and antimicrobial resistance have transformed nutritional strategies. The withdrawal of antibiotic growth promoters (**AGPs**) has further highlighted the need for dietary methods that promote gut health and nutrient efficiency.

Dietary protein supply has direct economic, environmental, and biological implications (Salahi et al., 2025). Insufficient protein causes impaired growth and efficiency, whereas excessive protein increases feed costs, N excretion, ammonia emissions, and waste of limited protein resources. As a result, modern poultry nutrition has shifted from maximizing CP inclusion to optimizing protein utilization. The concept of CP valorization—maximizing the useful value of dietary protein inputs while reducing losses—has become more prominent. Diets with reduced CP, based on digestible amino acids, are key to this strategy. This paper reviews the rationale and evidence supporting reduced-CP diets as a sustainable tool for poultry production without antibiotics.

### Nitrogen utilization efficiency in poultry

Poultry are relatively efficient users of dietary N compared with other livestock species, but substantial inefficiencies remain. In broilers, N retention is highest during early growth and lean tissue accretion, typically ranging from 50 to 66%, before declining with age (de Rauglaudre et al., 2023). In laying hens, N retention is lower (approximately 35–46%) but more stable across the production cycle due to its association with egg mass output (de Carvalho et al., 2012; Heo et al., 2023). Despite these efficiencies, a large proportion of dietary N is excreted, primarily as uric acid. Excess N contributes to ammonia volatilization, litter deterioration, and increased environmental footprint (Reddy and Krishna, 2009). Improving N utilization efficiency is therefore a major target in sustainable poultry nutrition, particularly in high-density production systems.

### Precision N nutrition and reduced CP diets

Precision N nutrition focuses on aligning dietary amino acid supply with animal requirements to minimize excess N intake. Conventional high CP diets often provide amino acids in excess of requirements due to the fixed amino acid profile of intact protein sources. These excess amino acids are deaminated and excreted, resulting in both economic losses and environmental burdens. Reduced CP diets mitigate this inefficiency by lowering total protein inclusion while supplementing limiting amino acids using crystalline sources (Baker, 2009). This approach reduces N intake without compromising growth (Hilliar et al., 2020) or egg production (de Carvalho et al., 2012; Heo et al., 2023), provided that amino acid balance and digestibility are properly managed. Advances in the availability of feed-grade amino acids have made this strategy increasingly practical in commercial poultry systems.

### Gastrointestinal health in AGP-free poultry systems

The gastrointestinal tract (**GIT**) of poultry is a complex and dynamic ecosystem involving interactions among diet, intestinal mucosa, and resident microbiota (Yadav and Jha, 2019). In AGP-free systems, nutritional management plays a critical role in maintaining gut integrity and microbial balance. Several factors disrupt intestinal health, including environmental stress, litter and water quality, dietary characteristics, anti-nutritional factors, toxins, and pathogenic microorganisms (Hilliar et al., 2020). Common indicators of compromised gut health include reduced growth rate, poor flock uniformity, wet litter, wet feces, feed passage, and abnormal cecal droppings. These symptoms are frequently associated with impaired nutrient digestion and microbial imbalance.

### Consequences of high CP diets on gut health

High CP diets pose specific challenges to intestinal health. Excess protein that escapes digestion in the upper gastrointestinal tract enters the lower intestine, where it undergoes microbial fermentation (Apajalahti and Vienola, 2016). Poultry derive little benefit from microbial protein synthesis, and instead experience negative consequences from protein fermentation.

Proteolytic fermentation promotes the proliferation of undesirable bacteria, including *Clostridium* spp., and results in the production of toxic metabolites such as ammonia, phenols, and branched-chain fermentation products (Rinttilä and Apajalahti, 2013). These compounds damage the intestinal mucosa, impair nutrient absorption, and increase endogenous energy losses associated with uric acid synthesis. High CP diets are also associated with increased litter moisture and ammonia emissions (Nahm, 2003; Collett, 2012), negatively affecting bird welfare by increasing the incidence of footpad dermatitis and related conditions (Martland, 1985).

### Reduced CP diets and production responses

**Broiler Chickens.** Experimental studies in broilers demonstrate that reducing dietary CP while supplementing essential amino acids significantly decreases excreta N concentration and moisture content (Alfonso-Avila et al., 2022; De Gula-Barrion et al., 2022; de Rauglaudre et al., 2023). Linear reductions in N excretion are consistently observed as dietary CP decreases, indicating improved N utilization efficiency. Importantly, these benefits can be achieved without compromising growth performance when amino acid ratios are adequately balanced. Reduced excreta moisture improves litter quality, reduces ammonia emission, and enhances bird welfare. These effects are particularly valuable in AGP-free systems, where gut health and litter management are critical determinants of performance.

**Laying Hens.** In laying hens, reduced CP diets have been shown to improve N utilization efficiency while maintaining energy utilization and productive performance (de Carvalho et al., 2012; Heo et al., 2023). When diets are formulated on a SID amino acid basis, reductions in CP do not negatively affect egg production or egg mass (Ali et al., 2025). These findings demonstrate that CP reduction is applicable beyond broiler production and can contribute to sustainability across poultry sectors.

### Protein quality, digestibility, and microbial fermentation

Protein quality and digestibility strongly influence the success of reduced CP diets. Poorly digestible or heat-damaged proteins increase the proportion of resistant protein reaching the lower intestine, providing substrate for microbial fermentation (Apalajahti and Vienola, 2016). Studies using low-digestibility protein sources, such as feather meal, demonstrate reduced N digestibility and increased fermentable protein residues (Elling-Staats et al., 2022). Protein source selection, appropriate processing, and the use of exogenous proteases can improve protein digestibility and reduce the flow of undigested protein to the hindgut (Gilbert et al., 2018). Improved digestibility limits microbial overgrowth, reduces *Clostridium* counts, and supports intestinal health, particularly in AGP-free production systems (Cardoso Dal Pont et al., 2020).

### Amino acid considerations in reduced CP diets

Production responses to reduced CP diets depend on both amino acid–related and non-amino acid–related factors (Kidd et al., 2021). Key amino acid considerations include accurate estimation of requirements, maintenance of ideal amino acid ratios, balance between essential and non-essential amino acids, and management of amino acid interactions.

Functional amino acids play an especially important role in reduced protein diets. Threonine is critical for mucin synthesis, immunoglobulin production, and maintenance of gut epithelial integrity, and may become limiting due to increased glycine demand (Adedokun and Olojede, 2019). Branched-chain amino acids (Val, Ile, and Leu) must be carefully balanced to avoid antagonisms, particularly in corn-based diets with relatively high leucine content (Ospina-Rojas et al., 2020). Arginine supports creatine synthesis, energy metabolism, and immune function through nitric oxide production and is essential in poultry due to the absence of a functional urea cycle (Fathima et al., 2023). Non-amino acid factors, including starch–protein digestion dynamics, dietary fiber level, phytate content, electrolyte balance, and total dietary fat, also influence responses to reduced CP diets and must be considered in formulation.

### Implications for sustainability and welfare

Reduced CP diets contribute to sustainability by lowering N excretion, ammonia emission, and reliance on high-protein feed ingredients such as soybean meal. Improved litter quality and reduced ammonia enhance bird welfare and working conditions in poultry houses. From an economic perspective, improved protein efficiency reduces feed costs per unit of output and buffers producers against volatility in protein ingredient prices. In AGP-free systems, reducing dietary protein also limits the availability of fermentable substrates for pathogenic bacteria, supporting gut health through nutritional rather than pharmaceutical means.

Precision N nutrition represents a practical and scientifically sound approach to improving sustainability in poultry production. Reduced CP diets, when formulated using digestible amino acid concepts and supported by high-quality protein sources, improve N utilization efficiency, support gut health, and reduce environmental impact without compromising performance. In AGP-free systems, this strategy also contributes to improved intestinal stability and bird welfare. Continued refinement of amino acid requirements, protein quality assessment, and feed formulation precision will further enhance the effectiveness of reduced CP diets in modern poultry production.

## Advances in analytical technology to support precision N nutrition

### Sanami Tatekura

In agriculture and livestock production, assessing the environmental impacts of products and practices is increasingly critical for climate and ecosystem health. A major concern is eutrophication in aquatic ecosystems, caused by excessive nutrient enrichment, primarily N and phosphorus (**P**), which promotes algal and plant overgrowth (Marine Water Information System for Europe, 2025). These algal blooms block sunlight and, upon decomposition by microorganisms, deplete oxygen and create hypoxic conditions that disrupt the ecosystem balance. The primary sources of these nutrients are runoff and leaching from fertilizers and manure in crop and livestock production. This underscores the need for strategies that minimize nutrient runoff and leaching through proper agricultural land management, as recommended by the Food and Agriculture Organization (Ongley, 1996), and for controlling N and P loading in agricultural activities. In feed formulation, adopting precision nutrition can further mitigate excess N loading while optimizing nutrient balance in the feed. Within the precision nutrition framework, it is essential to: (1) meet species-, age-, and production-specific nutritional requirements (Bailey, 2020; Lee and Rochell, 2022); (2) assess ingredient nutrient profiles to account for natural variability and select high-quality sources (Moss et al., 2021); (3) adjust formulations based on ingredient quality using the least-cost concept (Akintan et al., 2024); (4) optimize nutrient availability to enhance digestion and absorption, thereby improving feed efficiency (Bailey, 2020; Moss et al., 2021); and (5) reduce manure output and mitigate excess nutrient excretion, helping to minimize eutrophication and other environmental impacts (Bailey, 2020; Moss et al., 2021). Precision N nutrition involves formulating diets based on their N content, with growing emphasis on meeting amino acid requirements that vary across species and production stages. The least abundant essential amino acid in a diet often becomes the limiting factor for growth and performance. Combined with the inherent variability in amino acid profiles among and within ingredient types, this makes accurate characterization of feed ingredient amino acid composition essential. Leveraging this information enables the optimization of feed formulations to achieve an ideal amino acid profile, enhancing NUE while minimizing N excretion and waste.

### Integrating analytical technologies for precision nutrition

Achieving a balance between formulation precision and safety margins is essential to ensure nutritional adequacy, economic viability, and environmental sustainability (Shurson, 2019). While precise formulations are necessary to minimize nutrient excess and associated environmental impacts, it is equally critical to avoid deficiencies in essential nutrients, as these can compromise animal growth and performance. To address this challenge, advanced analytical technologies and decision-support tools should be integrated into the formulation process to accurately characterize the amino acid profiles of feed ingredients and identify sources of nutritional variability (Moss et al., 2021; Akintan et al., 2024). Such approaches enable effective adjustments to formulations, reducing the risk of productivity losses and nutrient deficiencies, while also preventing unnecessary costs associated with excessive supplementation of synthetic amino acids. Several analytical approaches are available for determining the nutrient composition of feed ingredients (Tahir et al., 2012; Mateos et al., 2019). Standard reference values published in feedstuff composition tables are widely used. However, their static nature often necessitates the application of large safety margins, which can increase formulation costs. Pre-established equations provide reliable estimates for plant-based ingredients but are less accurate for ingredients with high nutritional variability, such as animal protein by-products. The reference method, wet chemistry, offers high accuracy but is associated with drawbacks, including high costs, time-consuming sample preparation and analysis times, and susceptibility to sampling errors. Near infrared (**NIR**) spectroscopy serves as a secondary method to wet chemistry, requiring calibration against a primary method and relying on robust calibration models for quantification. However, NIR spectroscopy is considerably faster, more cost-effective than wet chemistry and enables simultaneous analysis of multiple nutrient parameters.

Accurate analysis of feed ingredient nutrient content is essential due to variability caused by factors such as geographic origin, processing, and storage (Moss et al., 2021). To manage these variations, it is critical to ensure supplier reliability and monitor ingredient quality through real-time assessments or historical trend analysis. NIR spectroscopy offers an efficient solution, enabling rapid, precise evaluation of numerous samples, which supports trend identification, enhances feed manufacturing control and creates opportunities to optimize formulations at lower cost. The working principle of NIR spectroscopy is based on the absorption of NIR light by organic molecular bonds in the sample, causing them to vibrate at characteristic frequencies that appear as distinct peaks in the resulting spectra. A calibration model, developed for specific feedstuffs, interprets these spectra to predict chemical composition. The accuracy of these predictions depends on the quality and diversity of samples used to build the calibration database, whereby the models establish correlations between NIR spectra and reference values, enabling reliable estimation of nutrient parameters in unknown samples (Burns and Ciurczak, 2001). Calibration models are therefore the critical component for quantifying nutrient content using NIR spectroscopy. AB Vista manages one of the largest NIR calibration databases in the animal feed industry, comprising over 900,000 global samples collected over 25 years. Through its online platform, Feed Quality Service (**FQS**), users can submit NIR spectra of feedstuff samples to obtain predictions for a wide range of nutrients, including 21 amino acid values and their digestibility values for poultry and swine across 15 feed ingredients. This globally representative database ensures robust calibration models with wide exploration ranges that can detect even minor variations between samples. Beyond accurate predictions, this capability provides valuable insights into ingredient behavior, supporting more informed feed formulation decisions.

### Nutrient profiling of major feed ingredients: corn and soya bean meal

Based on data collected from FQS, examples are provided to demonstrate the application of NIR spectroscopy in monitoring the amino acid profiles and characteristics of feed ingredients.

**Fig. 4:**
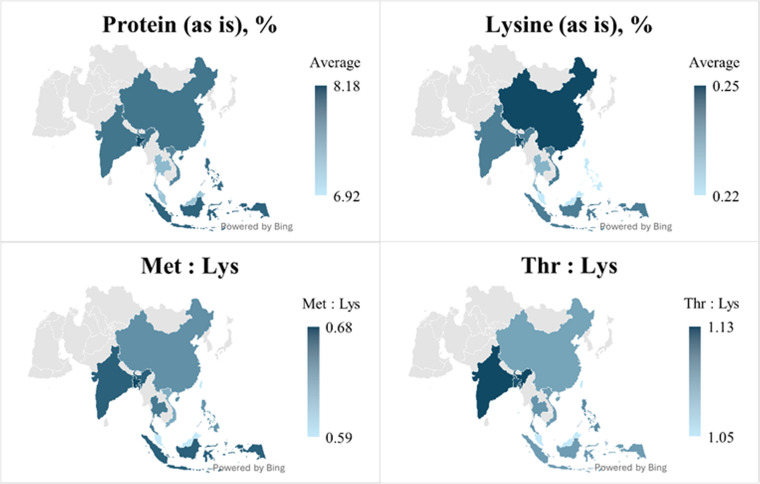
Regional variation in average protein and lysine content, and amino acid ratios of corn samples.

Corn is often overlooked in precision N nutrition due to its low protein content. However, because it is included in feed formulations at high inclusion rates, inaccurate nutrient estimates can significantly affect overall protein and amino acid levels and increase formulation costs. Table 3 presents the average protein, lysine, methionine, and threonine content of corn samples submitted to FQS in 2024 by country. Although corn is generally considered nutritionally stable, the data reveal notable regional variations (Fig. 4). For instance, corn used in the Philippines showed higher protein levels, but lower amino acid concentrations compared to other countries, while samples from China and India exhibited similar protein content yet differing amino acid ratios. These discrepancies indicate that standard reference values may not accurately reflect actual profiles. Given corn’s high inclusion rate in feed, even minor differences can significantly affect formulation cost. For example, methionine content ranging from 0.13% to 0.17% equates to approximately 220 g of DL-methionine per metric ton in a diet containing 55% corn. As synthetic amino acids are costly, precise estimation of amino acid content is essential.

**Table 3:** Average protein, lysine, methionine, and threonine content of corn samples^1^ by user country.

		Protein (as is)	Lysine (as is)	Methionine (as is)		Threonine (as is)	
Country	n	%	%	%	Met: Lys	%	Thr: Lys
**Bangladesh**	152	8.18	0.25	0.17	0.68	0.28	1.12
**Indonesia**	2498	7.97	0.24	0.16	0.67	0.26	1.08
**Philippines**	1339	7.89	0.22	0.14	0.64	0.24	1.09
**China**	888	7.83	0.25	0.16	0.64	0.27	1.08
**India**	3270	7.83	0.24	0.16	0.67	0.27	1.13
**Vietnam**	83	7.68	0.24	0.15	0.63	0.26	1.08
**Thailand**	2561	7.29	0.23	0.15	0.65	0.25	1.09
**Malaysia**	1566	7.20	0.22	0.13	0.59	0.23	1.05
**Taiwan**	86	6.92	0.22	0.13	0.59	0.23	1.05

n: number of samples.

^1^The AB Vista FQS database samples used in the analysis come from Bangladesh, China, India, Indonesia, Malaysia, the Philippines, Taiwan, Thailand, and Vietnam, between January 2024 and December 2024. Samples were corn (n = 12443), from all origins.

Soya bean meal is a key protein source in animal diets and requires monitoring for protein and amino acid profiles. Analyzing the soya bean meal samples submitted by users from 20 counties to FQS in 2024, a comparison across samples sourced from Brazil (=n= 12091), the United States (n= 3889), and Argentina (n= 1482) revealed an average protein content of 46.42%, 47.40%, and 47.58% respectively, with the largest difference of 1.16% between soya bean meal from Brazil and Argentina. Average amino acid patterns were generally consistent across origins, although Brazil (total % vs SID poultry %: lysine = 2.70 vs 2.50, methionine = 0.66 vs 0.57, threonine = 1.98 vs 1.49) showed slightly lower values, while the United States (total % vs SID poultry %: lysine = 2.77 vs 2.54, methionine = 0.68 vs 0.58, threonine = 2.04 vs 1.51) and Argentina (total % vs SID poultry %: lysine = 2.66 vs 2.57, methionine = 0.68 vs 0.59, threonine = 2.06 vs 1.54) were similar except for lysine. The largest gap between total and SID values occurred for threonine, with approximately 25%, indicating it may be a limiting factor in formulation and warrant supplementation or careful ingredient selection. Methionine, present at the lowest concentration, is highly sensitive to digestibility losses, making accurate estimation critical for nutrient availability. SID values provide a more accurate estimate of amino acid availability by correcting for basal endogenous losses. Using SID data helps prevent deficiencies, improves nutrient efficiency, and ensures animals receive the correct amino acid levels, supporting better N retention (Lee et al., 2017). However, total amino acid content remains important, particularly when evaluated alongside digestibility, as both are critical for assessing protein levels and soya bean meal processing quality (van Eys and Ruiz, 2021).

### Nutrient profiling of major feed ingredients: animal protein by-products

Animal protein by-products exhibit significant variability in protein, amino acid, and ash content due to differences in blend formulation, meat type, component ratios, rendering processes, and supplier standards. Cluster analysis is a valuable tool for identifying distinct groupings within such complex data sets. In nutrient profiling, it enables a clearer understanding of ingredient quality, supporting more targeted formulation adjustments. Given the complexity and volume of data, wet chemistry alone is impractical for such analysis. A diverse NIR calibration database covering multiple by-product types and sources offers a more efficient and reliable approach to characterizing ingredient quality (Burns and Ciurczak, 2001). In the following examples, K-means clustering combined with ANOVA was applied to FQS data to generate three nutrient-based clusters.

**Fig. 5:**
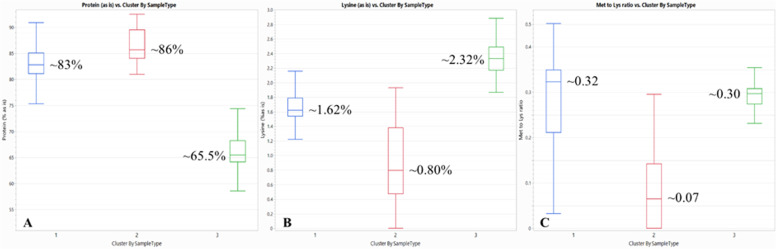
Three clusters for feather meal samples based on A: protein, B: lysine, and C: methionine-to-lysine ratio. The AB Vista FQS database samples used in the analysis come from China, Malaysia, the Philippines, Thailand, and Vietnam, between April 2023 and August 2025. Samples were feather meal (n = 568), from all origins. Samples were partitioned into three clusters, using K-means clustering of the variables’ moisture, ash, fat (acid hydrolysis), protein, lysine, methionine, threonine, arginine, and cysteine. Nutrient differences between clusters were assessed using ANOVA with Tukey’s HSD (*P* < 0.05) in JMP 18 (JMP Statistical Discovery LLC, Cary, NC).

The three clusters for meat and bone meal exhibited distinct nutrient profiles as shown in Table 4. Cluster 3 had higher methionine levels, suggesting possible inclusion of feather meal to boost protein content. Cluster 2 showed elevated ash and lower protein, typically indicating a greater proportion of bone. With only 19 samples in Cluster 2, further investigation to discern potential links to specific suppliers, customers, or regional sources could provide some valuable insights. Significant fluctuations in ingredient quality can hinder precise formulation, highlighting the need for continuous ingredient monitoring to anticipate changes and adapt formulations accordingly. Fig. 5’s box-and-whisker plots highlight clear nutritional differences among clusters for feather meal. Cluster 3 had the lowest protein but the highest lysine content, about three times greater than Cluster 2, which contained the highest protein levels. Methionine followed a similar trend, with Cluster 3 at 0.69%, Cluster 1 at 0.47%, and Cluster 2 at 0.08%. As shown in Fig. 5C, methionine-to-lysine ratios vary significantly, meaning no single ratio applies across all feather meal samples. Clusters 1 and 3 share similar ratios, although Cluster 1 is far more variable, increasing the risk of methionine deficiency when using feather meal from this group. Like the meat and bone meal example, Cluster 1′s variability warrants further investigation into sample origin. Overall, these clusters provide valuable insight into ingredient behavior.

**Table 4:** Average nutrient profiles (values in %) of the three clusters for meat and bone meal samples.

Cluster	Moisture	Ash (as is)	Fat AH (as is)	Protein (as is)	Lys (as is)	Met (as is)	Thr (as is)	Arg (as is)	Cys (as is)
**1 (**n **=740)**	5.00	30.72	11.39	51.71	2.21	0.67	1.55	3.45	0.55
**2 (**n **=19)**	7.26	40.25	16.04	33.30	1.56	0.22	0.84	2.03	0.17
**3 (**n **=556)**	4.51	25.09	11.20	56.48	2.68	0.88	1.79	3.66	0.53

n: number of samples.

^2^The AB Vista FQS database samples used in the analysis come from China, Malaysia, the Philippines, Thailand, and Vietnam, between April 2023 and August 2025. Samples were meat and bone meal (n= 1315), from all origins. Samples were partitioned into three clusters, using K-means clustering of the variables moisture, ash, fat (acid hydrolysis), protein, lysine, methionine, threonine, arginine, and cysteine. Nutrient differences between clusters were assessed using ANOVA with Tukey’s HSD (*P* < 0.05) in JMP 18 (JMP Statistical Discovery LLC, Cary, NC).

Traditionally, proximate values have guided feed formulation adjustments, but amino acid data enables finer optimization for better N retention and NUE. Previously, amino acid values were based on calculations and reference ratios; however, NIR calibration models now allow rapid, cost-effective prediction of amino acid content. A robust and diverse NIR calibration database further improves the detection of nutritional variability. While the examples provided reflect global or regional analyses, similar evaluations can be performed for individual customers. Using NIR data, cluster analysis can differentiate ingredients by nutrient quality, enabling the creation of multiple ingredient lots, typically two or three, for separate use in feed formulation. This approach allows formulation matrices to be adapted according to lot quality, achieving precise nutrition across varying batches and suppliers.

## Conclusion

Adopting precision N nutrition—defined by reducing dietary CP and precisely supplementing SID amino acids—is a key strategy for improving the efficiency, welfare, and environmental sustainability of broiler production. Research indicates that CP levels can be reduced to approximately 16% during the 7-21 days of age without compromising growth performance, provided that all essential amino acids and non-essential Gly_equiv_ are carefully balanced to meet genetic potential. This nutritional approach reduces the harmful effects of indigestible protein on gut health, conserves metabolic energy by decreasing uric acid synthesis, and significantly lowers N excretion, water consumption, and the global warming potential of poultry farming. While effective implementation requires advanced analytical tools and careful maintenance of dietary electrolyte balance, precision nutrition effectively decouples bird performance from the negative environmental and physiological impacts of traditional high-protein diets.

## CRediT authorship contribution statement

**Jae Cheol Kim:** Writing – review & editing, Writing – original draft, Conceptualization. **Behnam Saremi:** Writing – review & editing, Writing – original draft. **Wölfgang Siegert:** Writing – review & editing, Writing – original draft. **Rommel Sulabo:** Writing – review & editing, Writing – original draft. **Sanami Tatekura:** Writing – review & editing, Writing – original draft.

## Disclosures

The authors declare no conflicts of interest.
